# A Randomized Controlled Trial of Storytelling as a Communication Tool

**DOI:** 10.1371/journal.pone.0077800

**Published:** 2013-10-25

**Authors:** Lisa Hartling, Shannon D. Scott, David W. Johnson, Ted Bishop, Terry P. Klassen

**Affiliations:** 1 Alberta Research Centre for Health Evidence, Department of Pediatrics, University of Alberta, Edmonton, Alberta, Canada; 2 Faculty of Nursing, University of Alberta, Edmonton, Alberta, Canada; 3 Departments of Pediatrics and Physiology & Pharmacology, University of Calgary, Calgary, Alberta, Canada; 4 Department of English and Film Studies, University of Alberta, Edmonton, Alberta, Canada; 5 Manitoba Institute for Child Health, Winnipeg, Manitoba, Canada; Brunel University, United Kingdom

## Abstract

**Introduction:**

Stories may be an effective tool to communicate with patients because of their ability to engage the reader. Our objective was to evaluate the effectiveness of story booklets compared to standard information sheets for parents of children attending the emergency department (ED) with a child with croup.

**Methods:**

Parents were randomized to receive story booklets (n=208) or standard information sheets (n=205) during their ED visit. The primary outcome was change in anxiety between triage to ED discharge as measured by the State-Trait Anxiety Inventory. Follow-up telephone interviews were conducted at 1 and 3 days after discharge, then every other day until 9 days (or until resolution of symptoms), and at 1 year. Secondary outcomes included: expected future anxiety, event impact, parental knowledge, satisfaction, decision regret, healthcare utilization, time to symptom resolution.

**Results:**

There was no significant difference in the primary outcome of change in parental anxiety between recruitment and ED discharge (change of 5 points for the story group vs. 6 points for the comparison group, p=0.78). The story group showed significantly greater decision regret regarding their decision to go to the ED (p<0.001): 6.7% of the story group vs. 1.5% of the comparison group strongly disagreed with the statement “I would go for the same choice if I had to do it over again”. The story group reported shorter time to resolution of symptoms (mean 3.7 days story group vs. 4.0 days comparison group, median 3 days both groups; log rank test, p=0.04). No other outcomes were different between study groups.

**Conclusions:**

Stories about parent experiences managing a child with croup did not reduce parental anxiety. The story group showed significantly greater decision regret and quicker time to resolution of symptoms. Further research is needed to better understand whether stories can be effective in improving patient-important outcomes.

**Trial Registration:**

Current Controlled Trials, ISRCTN39642997 (http://www.controlled-trials.com/ISRCTN39642997)

## Introduction

Children’s illness and injury cause parental anxiety[1,2]; major sources of parental anxiety are uncertainty about the condition and its management[3,4]. Providing information about the illness and processes of care has been linked with reduced anxiety and uncertainty as well as greater satisfaction with medical services and more appropriate healthcare utilization[2,4]. Standard written instructions have been found lacking[5], while alternative formats (such as video presentations, illustrations, or cartoons) have been found to be more effective. Storytelling is one of the oldest forms of communication[6]. Recently, there has been resurgence in the use of storytelling in medicine in diagnostics[7,8], therapeutics[9-11], and education[12-17]. Stories may be effective because they are easy to understand and more likely to be remembered[18]. An appeal of storytelling is its ability to present information couched within a personal account that engages the reader and validates their own experiences[19,20]. Few randomized trials have evaluated the effectiveness of stories and among the trials that exist, there is variation in the purpose of the stories and target populations. Only one trial involved a pediatric, clinical population and addressed management of parental anxiety. Melnyk et al. developed an educational-behavioural intervention for children admitted to a pediatric intensive care unit and their mothers which included reading and discussing a story about a young child who successfully copes with a stressful hospitalization[21]. While there were no differences in parental anxiety during hospitalization, the intervention group showed reduced anxiety at 1 month post-discharge (effect size 0.32), as well as reduced depression, and symptoms of post-traumatic stress disorder following hospitalization. The primary aim of this study was to investigate the effectiveness of storytelling compared to standard information sheets for parents of children attending the emergency department (ED) with a child with croup. Croup was chosen as the condition with which to examine the hypothesis because of the frequency of its presentation to the ED[22-24], the anxiety that it causes for parents[25], and the large body of evidence that supports the therapeutic management of the disease[26].

## Methods

**Figure 1:**
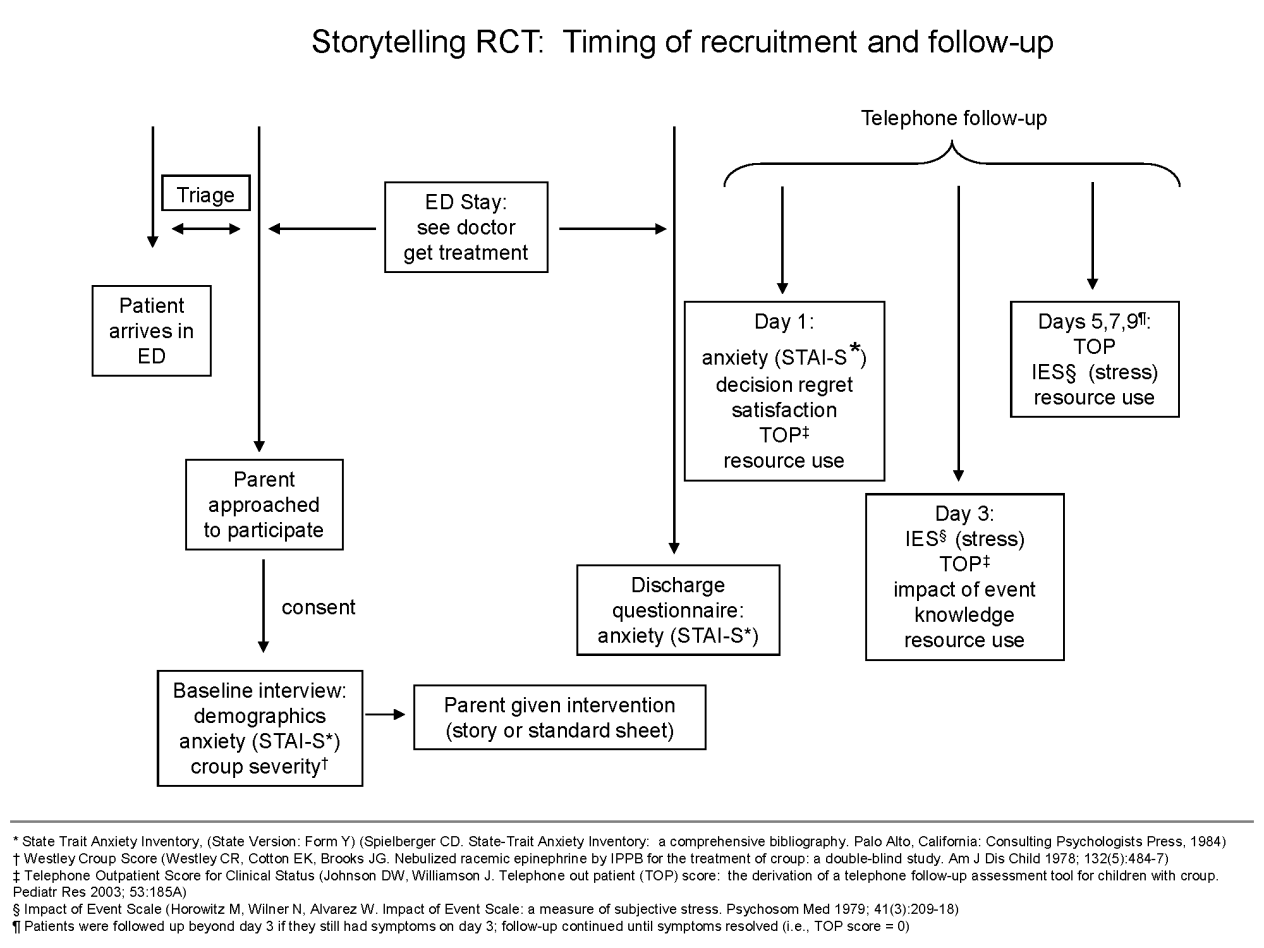
Flow diagram of patient recruitment and follow-up

The protocol for this trial and supporting CONSORT checklist are available as supporting information; see Checklist S1 and protocol S1. This parallel randomized trial was conducted in the emergency departments of three tertiary care pediatric hospitals in Canada: Stollery Children’s Hospital (SCH) in Edmonton, Alberta Children’s Hospital (ACH) in Calgary, and the Children’s Hospital of Eastern Ontario (CHEO) in Ottawa. The original study protocol included only two sites (SCH and ACH); CHEO was added in the last year of the study in order to complete recruitment in a timely manner. Recruitment occurred between October 2007 and March 2010. The study was registered (http://www.controlled-trials.com/ISRCTN39642997) and approved by the respective institutional Ethics Review Boards prior to commencement: the University of Alberta Health Research Ethics Board, the University of Calgary Conjoint Health Research Ethics Board, and the CHEO Research Ethics Board. Study subjects provided written informed consent prior to participation; the consent process and forms were approved by the ethics committees. Parents of children with a clinical diagnosis of croup were eligible. Parents had to meet the following additional criteria: 1) have a telephone and agree to be contacted for follow-up interviews; 2) fluent in English; 3) provide informed consent; 4) no prior visit to an ED during this episode of the disease; 5) no prior visit to an ED for another episode of croup during the study period. Parents were excluded if: 1) stridor was due to another cause; 2) parent had previously participated in the study. The experimental intervention was a package containing three booklets that integrated stories, as told by parents of children with croup attending the ED, with evidence regarding the epidemiology and treatment of the condition. Each story reflected a case of different severity (mild, moderate, severe). The purpose of the stories was to deliver health information to the parents in the context of real parent experiences. We hypothesized that stories would generate more impact than standard information sheets because of the situation-specific details and human experience related through the recounting of events[19]. Development of the story booklets has been described[27]. A standard information sheet produced by the Alberta Medical Association was the control intervention. After they had been randomized to treatment groups, parents received the story package or the information sheet. In addition, all children received usual medical care. The interventions are available elsewhere[28]. While we planned to recruit parents as soon as possible after arriving at the ED, due to practical and confidentiality issues we were unable to recruit parents when they first arrived at the ED; therefore, recruitment occurred once the child and parent were in a treatment room. The randomization sequence was computer generated by a statistician. After obtaining informed, written consent from the parent, the research personnel opened the next envelope in a series of consecutively labeled, sealed, opaque envelopes. The research personnel and treating physician were unaware of the next group assignment. Parents were blind to the interventions being compared. While they were aware that the study was evaluating some aspect of managing croup, they did not know what aspect was being tested. Because of the nature of the intervention, the researcher and other ED personnel were not blind to the intervention that participants received. Figure 1 describes recruitment and follow-up. Immediately after recruitment and informed consent, the research personnel collected demographic information and participants completed the State Trait Anxiety Inventory (STAI)[29]. The research personnel assessed the severity of the child’s illness using the Westley Croup Score[30]. On discharge from the ED, participants completed the STAI again. The research personnel contacted the parent by telephone at 1 day and 3 days after the ED visit. Parents of children who were still symptomatic at day 3 were contacted every 2 days until the symptoms resolved or until day 9.

*The primary outcome* was change in parental anxiety from baseline (immediately following recruitment to the study) to discharge from the ED. State anxiety was measured using the Spielberger STAI which is a well-known instrument designed to measure state anxiety at the time of administration, in the recent past, or at a future point in time. The inventory consists of 20 items that ask respondents to indicate how much each statement reflects how they feel on a 4-point Likert scale ranging from “not at all” to “very much so”. Scores are summed and range from 20 to 80; higher scores indicate higher anxiety[29]. The scale has good internal consistency and takes 6-10 minutes to complete during initial administration and less than 5 minutes during repeat administrations[29]. Secondary outcomes are described in Table 1. 

**Table 1:** Secondary Outcomes.

**Outcome**	**Method of assessment**	**Timing of assessment post emergency department visit**
Expected future anxiety	State-Trait Anxiety Inventory regarding expected anxiety should they face another incident with croup in the future [29]	Day 1
Event impact	The Impact of Event Scale includes 15 self-report items to measure intrusion (7 items) and avoidance (8 items) resulting from exposure to anxiety-producing events (in this case, the child’s croup illness). Using a 4-point Likert scale ranging from “not at all” to “often,” respondents indicated how frequently the items were relevant to them during their child’s illness with croup. This tool has been shown to have good internal consistency and takes up to 10 minutes to complete[53].	Last follow-up (day 3, 5, 7, or 9 depending on when croup symptoms resolved)
Parental knowledge	10 questions developed specifically for this study about the natural history of the disease, symptoms, and management strategies	Day 3
Parental satisfaction	Independent questions with responses on a 5-point Likert scale for satisfaction with overall ED visit and with the information they received	Day 1
Parental decisional regret	Validated scale assesses “remorse or distress over a decision”[54] (i.e., the decision to take their child to the ED). Participants rate five statements from strongly agree to strongly disagree (see Table 6 for specific items).	Day 1
Incidence of return to be evaluated by a physician (or other health care practitioner) for croup	Parent self-report	Assessed at each follow-up interview
Healthcare utilization	Parent self-report of seeking further medical care for croup following the visit to the ED, the type of consultation, location of care, type of care provider, and whether they were prescribed any medication	Assessed at each follow-up interview
Resource utilization	Parent self-report regarding costs for medication, equipment (e.g., humidifiers), parking and travel, ambulance service, child care, and time lost to usual activities	Assessed at each follow-up interview
Ongoing croup symptoms	Telephone Outpatient Score for Clinical Status (TOP score)[55,56]. The TOP score involves three questions dealing with croup symptoms: whether the child makes a noise when breathing, whether the child has a cough, and whether or not the cough is barky.	Assessed at each follow-up interview
Long-term outcomes	knowledge of croup, other incidents of croup occurring after the study, and their reactions to and management of subsequent croup episodes	1 year

## Sample Size

The initial anxiety level in both groups was estimated to be 45 on the STAI-S[1,4,31-38]. Based on previous research[21], we hypothesized that parents in the story group would return to a “normal” level of anxiety following treatment (i.e., 36 or 37)[29], while those in the comparison group would remain more anxious (i.e., 39 or 40)[29]. We conducted sample size calculations, using a two-sided, two sample t-test with a significance level of 0.05 and standard deviation of 10 (based on the cited studies), to detect a difference of 3 points. For 80% power, we required 176 individuals per group. The sample size was inflated by 20% (210 per group) to account for potential contamination and drop-outs[39]. 

### Data analysis

Baseline variables were described for each group. For the primary outcome, a change score from baseline to discharge was calculated for each patient. The median change scores were compared between groups using the Mann-Whitney test. Continuous outcomes were compared between study groups using independent-groups t-tests if the data were normally distributed, and the Mann-Whitney test if the data were skewed. Categorical outcome data were analyzed using the Chi-square test. Kaplan-Meier curves for time to resolution of symptoms were tested for equality using the log rank (Mantel-Cox) test. Our analysis was based on intention-to-treat where all participants who were randomly assigned to a study group were included whether or not they received or complied with (i.e., read) the intervention to which they were assigned. Analyses were conducted using SPSS 18. 

Study data and additional analyses not reported here are available from the corresponding author.

## Results

**Figure 2:**
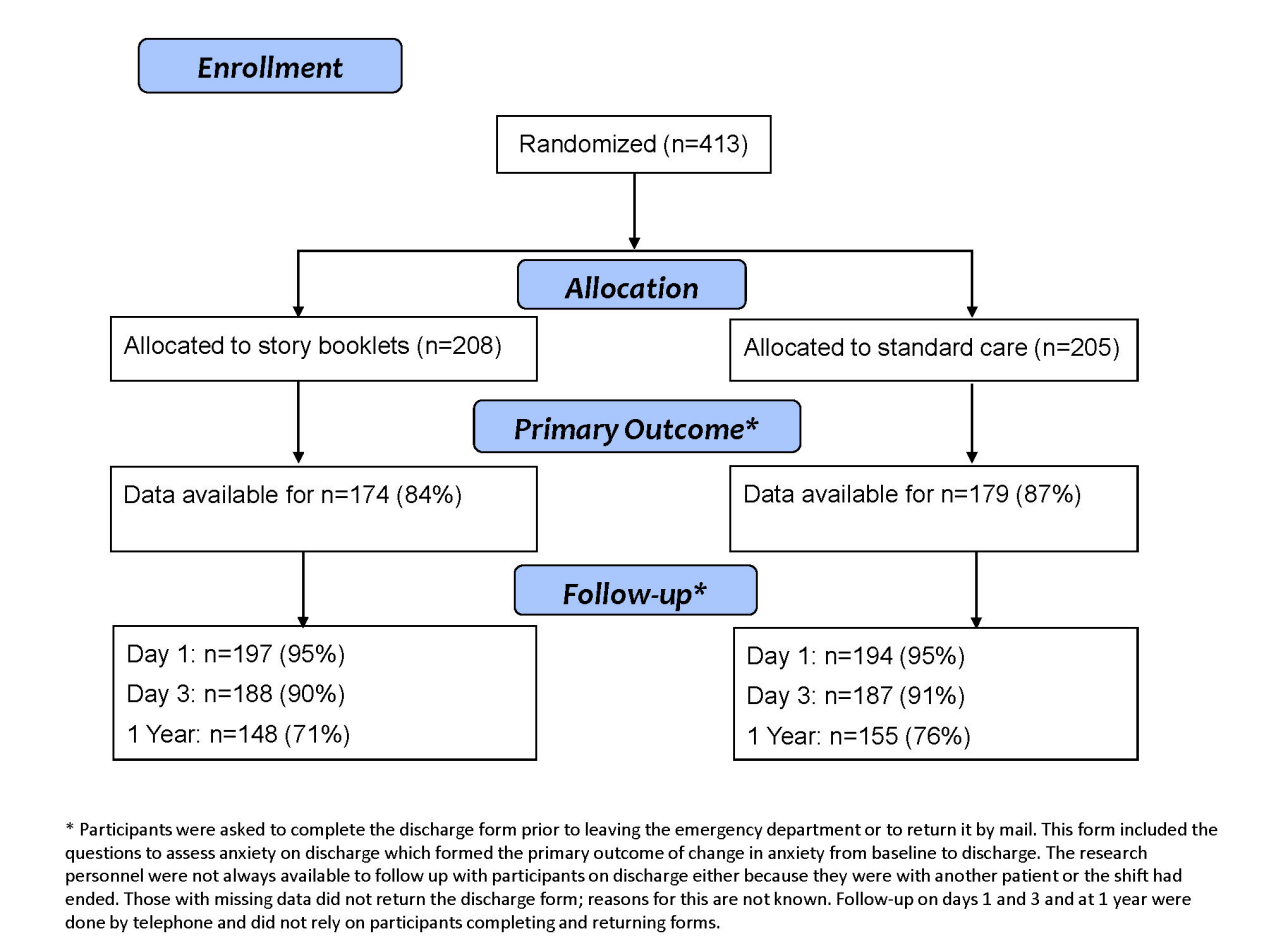
Recruitment and follow-up of study participants.

413 parents participated. Figure 2 describes the recruitment and follow-up of study participants to day 3 which was the last follow-up point required for all participants. There were no notable differences between groups in demographic variables (Table 2).

**Table 2:** Demographics.

	**Story booklets**		**Information sheet**	
	**N=208**		**N=205**	
	**n**	%*	**n**	%*
**Age of participant (i.e., parent), years (mean, SD)**	34.4	6.1	33.3	6.4
**Sex of participant (i.e., parent)**				
Female	164	78.8	172	83.9
Male	43	20.7	29	14.1
Unknown	1	0.5	4	2.0
**Age of child, years (median, IQR)**	2.1	1.2,3.5	1.9	1.2,3.1
**Site**				
Alberta Children's Hospital	92	44.2	91	44.4
Stollery Children's Hospital	96	46.2	93	45.4
Children’s Hospital of Eastern Ontario	20	9.6	21	10.2
**Number adults living in the home**				
1	9	4.3	14	6.8
2	174	83.7	162	79.0
>2	24	11.5	29	14.1
**Number adults participating in care of child**				
1	6	2.9	10	4.9
2	169	81.3	158	77.1
>2	32	15.4	37	18.0
**Relationship to child**				
Parent	204	98.1	200	97.6
Other	2	1.0	4	2.0
**Education**				
grades 1-9	1	0.5	1	0.5
grades 10-11 (some high school)	9	4.3	9	4.4
high school graduate	25	12.0	42	20.5
some college/university	37	17.8	38	18.5
college graduate	64	30.8	60	29.3
post-graduate education or degree	68	32.7	52	25.4
**Marital status**				
never married	7	3.4	15	7.3
married/common-law	187	89.9	174	84.9
separated, divorced, or widowed	11	5.3	14	6.8
Other	1	0.5	1	0.5
**Household income (Canadian $)**				
<15,000	7	3.4	7	3.4
15-29,000	10	4.8	11	5.4
30-44,000	15	7.2	12	5.9
45-59,000	15	7.2	19	9.3
60-74,000	26	12.5	15	7.3
75-90,000	16	7.7	31	15.1
>90,000	93	44.7	87	42.4
NR	25	12.0	23	11.2
**Ethnic or minority group**				
No	164	78.8	153	74.6
Yes	37	17.8	45	22.0
**Place of birth**				
North America	155	74.5	168	82.0
Outside of North America	47	22.6	32	15.6

^*^percentages except for age where value reported is a standard deviation

SD=standard deviation; IQR=inter-quartile range SD: standard deviation; IQR: inter-quartile range; NR: no response

Overall, parents demonstrated a moderate level of concern at baseline with a mean self-rating of 6.3 (SD 2.5) on a scale of 1 to 10 (10 is highest concern) (Table 3). The majority of participants had no prior history with their children of croup admissions, ICU admissions, or intubations (Table 4). 

**Table 3:** Parental concern at baseline.

	**Story booklets**		**Information sheet**	
	**N=208**		**N=205**	
	**n**	**%**	**n**	**%**
**Level of concern about the following items:**				
uncomfortable aspect of child's cough				
0 (not at all)	7	3.4	2	1.0
1	46	22.1	29	14.1
2	80	38.5	105	51.2
3 (extremely)	74	35.6	69	33.7
NR	1	0.5	0	0
unusual sound or nature of the cough				
0 (not at all)	12	5.8	4	2.0
1	42	20.2	43	21.0
2	75	36.1	85	41.5
3 (extremely)	78	37.5	73	35.6
NR	1	0.5	0	0
unusual sound of child's breathing				
0 (not at all)	9	4.3	5	2.4
1	34	16.3	22	10.7
2	70	33.7	78	38.0
3 (extremely)	95	45.7	100	48.8
NR	0	0	0	0
effort that child is making to breathe				
0 (not at all)	17	8.2	12	5.9
1	37	17.8	37	18.0
2	65	31.3	71	34.6
3 (extremely)	89	42.8	85	41.5
NR	0	0	0	0
child is not getting enough oxygen				
0 (not at all)	39	18.8	37	18.0
1	46	22.1	43	21.0
2	68	32.7	66	32.2
3 (extremely)	54	26.0	57	27.8
NR	1	0.5	2	1.0
child may be wheezing or have asthma				
0 (not at all)	49	23.6	42	20.5
1	34	16.3	54	26.3
2	69	33.2	53	25.9
3 (extremely)	55	26.4	55	26.8
NR	1	0.5	1	0.5
child's sleep was disturbed				
0 (not at all)	27	13.0	24	11.7
1	43	20.7	34	16.6
2	65	31.3	73	35.6
3 (extremely)	73	35.1	72	35.1
NR	0	0	2	1.0
parent felt increasingly tense or frustrated as a result of the illness				
0 (not at all)	40	19.2	34	16.6
1	52	25.0	62	30.2
2	66	31.7	74	36.1
3 (extremely)	50	24.0	35	17.1
NR	0	0	0	0
child might be hospitalized				
0 (not at all)	42	20.2	50	24.4
1	63	30.3	71	34.6
2	57	27.4	50	24.4
3 (extremely)	46	22.1	32	15.6
NR	0	0	2	1.0
illness might recur in the future				
0 (not at all)	15	7.2	19	9.3
1	54	26.0	50	24.4
2	69	33.2	69	33.7
3 (extremely)	70	33.7	67	32.7
NR	0	0	0	0
not knowing about this illness				
0 (not at all)	34	16.3	36	17.6
1	62	29.8	64	31.2
2	57	27.4	60	29.3
3 (extremely)	53	25.5	43	21.0
NR	2	1.0	2	1.0
**Overall concern (scale 1-10**) **(mean, SD)**	6.0	2.6	6.4	2.3

NR=no response; SD=standard deviation

**Table 4:** History of previous illness, severity of illness at baseline, and ED visit.

**History**	**Story booklets**		**Information sheet**	
	**N=208**		**N=205**	
	**n**	%*	**n**	%*
**Parent first noticed respiratory symptoms** (number of days to ED visit) (median, IQR)	1	0,2	1	1,2
**Prior history of croup**				
no history	115	55.3	121	59.0
History same child	38	18.3	35	17.1
History other child	27	13.0	26	12.7
History both	26	12.5	22	10.7
**Prior history of croup admissions**				
no admits	183	88.0	176	85.9
ED visit only this child	9	4.3	9	4.4
ED visit only other child	3	1.4	8	3.9
previous admissions this child	5	2.4	5	2.4
previous admissions other child	7	3.4	6	2.9
**Prior admissions to ICU**				
no ICU admits	204	98.1	200	97.6
ICU this child	1	0.5	2	1.0
ICU other child	1	0.5	1	0.5
**Prior intubations**				
no history	189	90.9	180	87.8
History this child	9	4.3	14	6.8
History other child	8	3.8	8	3.9
History both	1	0.5	2	1.0
**Prior serious illness or chronic medical condition this child**				
No	166	79.8	158	77.1
Yes	41	19.7	46	22.4
**Croup severity**				
total score (median, IQR)	1	0,3	2	1,3
0	58	27.9	39	19.0
1	56	26.9	50	24.4
2	31	14.9	49	23.9
3	29	13.9	30	14.6
4	15	7.2	25	12.2
5	9	4.3	8	3.9
>5	5	2.4	3	1.5
missing	5	2.4	1	0.5
**ED Care**				
*Disposition*				
left without being seen	1	0.5	4	2.0
discharged home	185	88.9	187	91.2
Admitted	8	3.8	7	3.4
Other	0	0	1	0.5
*Prior to recruitment patient seen by*				
triage nurse	198	95.2	197	96.1
staff nurse	107	51.4	116	56.6
Resident	45	21.6	42	20.5
staff physician	70	33.7	59	28.8
Other	6	2.9	7	3.4
*Prior to recruitment treatment ordered*				
Yes	149	71.6	151	73.7
No	46	22.1	49	23.9
*Read information during ED visit*	N=173		N=179	
Read study material	101	58.4	127	70.9
Read additional information	43	24.7	33	18.4

percentage except where otherwise indicated

ED=emergency department; IQR=interquartile range; ICU=intensive care unit

The majority of patients presented with mild croup: median croup score of 2 (IQR 1,3) on a scale of 0 to 17 (Table 4). Approximately 90% of the patients were discharged home from the ED; less than 5% were admitted. Prior to recruitment approximately 1 in 5 children had been seen by the staff physician and treatment had been ordered for almost 70%. 

Approximately 65% of participants read the study material during their ED stay while 22% read additional information on croup. Fewer parents in the story group read the study material (58% versus 71%) but more parents in the story group read additional material (25% versus 18%). There was no association between parental education level and whether they read the stories or sought additional information.

The baseline anxiety score on the STAI was 37.2 (SD 12.3) for the story group versus 38.8 (SD 12.3) for the comparison group (Table 5). At discharge the STAI scores were approximately 5 to 6 points lower for both groups (32.2 and 32.8, respectively), with no significant difference between groups in change in parental anxiety from baseline to discharge (p=0.78). 

**Table 5:** Comparison of primary and secondary outcomes.

**Outcome**	**Story booklets**	**Information sheets**	**P-value**
**Anxiety - STAI**			
Baseline (mean, SD)	38.1 (12.1)	38.5 (11.4)	0.71
Discharge (mean, SD)	33.1 (11.1)	32.7 (9.3)	0.72
Discharge-Baseline (median, IQR)	4.1 (-1,10)	5.4 (0,10)	0.24
**Expected anxiety in future episodes of croup (mean, SD)**	42.0	43.4	0.28
**Decision Regret** (mean, SD)	1.24 (0.42)	1.16 (0.29)	0.04
**Satisfaction**			
Expectations for treatment and care (n)			0.44
very satisfied	132 (63.5)	136 (66.3)	
somewhat satisfied	48 (23.1)	47 (22.9)	
neither satisfied nor dissatisfied	4 (1.9)	3 (1.5)	
somewhat dissatisfied	7 (3.4)	7 (3.4)	
very dissatisfied	6 (2.9)	1 (0.5)	
Expectations for Information (n)			0.42
very satisfied	147 (70.7)	136 (66.3)	
somewhat satisfied	37 (17.8)	47 (22.9)	
neither satisfied nor dissatisfied	4 (1.9)	3 (1.5)	
somewhat dissatisfied	3 (1.4)	4 (2.0)	
very dissatisfied	2 (1.0)	0 (0)	
**Knowledge** (mean, SD)	8.6 (1.6)	8.5 (1.3)	0.55
Impact of event scale*			
Intrusion sub-scale (median, IQR)	6 (1,12)	6 (1,13.5)	0.20
Avoidance sub-scale (median, IQR)	3 (0,7)	3 (0,6.5)	0.51
Total (median, IQR)	10 (3,19)	10 (4,21)	0.80

STAI=State Trait Anxiety Inventory; IQR=interquartile range; SD=standard deviation; ED=emergency department; NR=not reported

* Intrusion (e.g., unbidden thoughts, troubled dreams^45^ p=0.20); avoidance (e.g., denial of event importance, blunted sensation^45^).

Expected future anxiety showed no significant differences between groups (42.0 versus 42.6, p=0.36). Interestingly, expected future anxiety was substantially higher than the participants’ baseline anxiety (Table 5).

The impact resulting from exposure to anxiety-producing events showed no significant differences between groups overall (median=10 for both groups, p=0.80) or for the two subscales (intrusion and avoidance, Table 5). 

There was no significant difference in knowledge between groups at day 3 (8.6 versus 8.4, p=0.5). Overall, the knowledge level was high for both groups: mean of 8.5 (SD 1.5) out of 10.

The majority of patients in both groups (64% and 68% respectively) were “very satisfied” with the treatment and care they received in the ED. A further 19% and 21% were “somewhat satisfied.” The results for satisfaction around their expectations for information were similar with the majority “very satisfied” (77% and 71%) or “somewhat satisfied” (17% and 21%). There was no significant difference between groups in satisfaction.

The mean regret score, assessed at 1 day post-ED visit, was significantly higher in the story group compared to the comparison group (1.3 versus 1.2; t-test, p=0.04). When the five items in the regret scale were assessed independently, only one item showed a significant difference between groups (Table 6). More parents in the story group showed less agreement with the statement “I would go for the same choice if I had to do it again” (p=0.017). 

**Table 6:** Comparison of decision regret scale*.

**Statements in Decision Regret Scale**	**Story booklets**	**Information sheets**	**P-value**
	N=208	N=205	
It was the right decision.			0.39
Strongly agree	149 (71.6)	160 (78.0)	
Agree	42 (20.2)	31 (15.1)	
Neither agree nor disagree	3 (1.4)	3 (1.5)	
Strongly disagree	3 (1.4)	1 (0.5)	
I regret the choice that was made.			0.41
Strongly agree	4 (1.9)	1 (0.5)	
Agree	0 (0)	1 (0.5)	
Neither agree nor disagree	6 (2.9)	7 (3.4)	
Strongly disagree	186 (89.4)	185 (90.2)	
I would go for the same choice if I had to do it over again.			0.02
Strongly agree	133 (63.9)	151 (73.7)	
Agree	38 (18.3)	30 (14.6)	
Neither agree nor disagree	12 (5.8)	9 (4.4)	
Strongly disagree	14 (6.7)	3 (1.5)	
The choice did my child a lot of harm.			0.61
Strongly agree	0 (0)	0 (0)	
Agree	2 (1.0)	2 (1.0)	
Neither agree nor disagree	3 (1.4)	1 (0.5)	
Strongly disagree	192 (92.3)	191 (93.2)	
The decision was a wise one.			0.71
Strongly agree	156 (75.0)	151 (73.7)	
Agree	37 (17.8)	42 (20.5)	
Neither agree nor disagree	1 (0.5)	1 (0.5)	
Strongly disagree	3 (1.4)	1 (0.5)	

* Decision Regret (measured at day 1 or 3 post-ED visit): Parents were asked to respond to the statements regarding their decision to take their child to the ED for the episode of croup in question.

There was no difference between groups in the number of participants who returned to a physician or the ED (27.9% versus 24.5%, p=0.44), or in the incidence of contacting a healthcare professional following the ED visit (28.9% stories versus 24.5% comparison, p=0.36). 

The survival distributions of time to no symptoms (TOP score=0) were significantly different (mean 3.7 days stories vs. 4.0 days comparison, median 3 days both groups; log rank test, p=0.04). A greater proportion of the story group reported no symptoms by day 1 (18.4% versus 14.4%) while a greater proportion of the control group reported symptoms until day 9 (5.1% vs. 1.5%); further, five patients in the control group reported ongoing symptoms at day 9.

303 parents (73.3%) responded to the 1-year follow-up. Average knowledge scores were less than immediately following the ED visit (mean 7.11, SD 1.77); however, there was no significant difference between groups (mean 7.20 [SD 1.77] stories vs. 7.03 [1.77] comparison). Overall, 30.7% of parents reported at least one additional episode of croup in the same or another child after the study episode (26.4% stories, 34.8% comparison). When questioned about their confidence that they knew what to do when the subsequent croup episodes occurred, 82% were very confident, 15% somewhat confident, and 2% not at all confident. While there was no significant difference between groups, both parents who reported being not at all confident were in the comparison group.

## Discussion

This is one of few randomized trials examining stories to communicate with healthcare consumers. As this trial evaluated a novel intervention that has not been the focus of previous research, we explored a number of outcomes; however, only two notable differences were found. First, parents in the story group showed greater decision regret. After reading the stories parents may have felt that they could have managed at home and avoided the trip to the ED. Second, parents in the story group reported resolution of croup symptoms earlier. Children’s symptoms may have resolved more quickly due to how the parents managed the child at home. Alternatively, the parents’ *perception* of the child’s symptoms may have been affected by the intervention. For instance, if the parents who read the stories felt more reassured or more confident in their knowledge or ability to manage the condition, they may have been less bothered by ongoing coughing or other symptoms. Further, by reading accounts of how croup resolves in the stories, parents may have been more aware of the natural progression of the disease[40].

We found no significant difference for the remaining outcomes including the primary outcome of change in anxiety between study enrolment and ED discharge. One of the main reasons for this finding may be related to the clinical context. While croup may cause heightened anxiety when the child first exhibits symptoms, or exhibits the most intense symptoms, the medical treatment is highly effective with very rapid results, the condition is transient, and there are no known ongoing or long-term effects of either the condition or the treatment. Therefore, large differences may be unexpected regardless of the intervention. The baseline anxiety observed in our study showed that parents were within “normal” levels of state anxiety[29]. This may have been related to the timing of recruitment: due to practical and confidentiality issues we were unable to recruit parents when they first arrived at the ED therefore many participants had already been seen by a healthcare professional and in some cases had already received treatment. A previous study identified the loss of parental roles as a major source of stress[21]. This may be more applicable to conditions requiring highly intensive medical interactions or where the child is removed from the parent for periods of time. 

Other studies of similar interventions have attributed a lack of significant difference to the fact that the control group also received an intervention that may have resulted in decreased anxiety[21,41]. A “pure” control group receiving no patient education materials may have demonstrated a greater effect of the test intervention. Participants in this study received interventions which overlapped in terms of content thereby minimizing the relative impact of one intervention over the other. A “pure” control group (i.e., placebo arm with no handout) should be considered in future research to establish whether educational material of any form has an effect.

The choice of outcomes in our study was based on factors that could be easily measured and quantified. Stories may have a stronger or more consistent effect on factors that are more challenging to measure. We hypothesized that the stories would provide greater overall comfort to the parents (or attend to their emotional reactions), although this construct was difficult to define and quantify. Therefore, we chose a variety of outcomes that we felt were related to this construct in different ways including anxiety, satisfaction, decision regret, and impact of the event. Many of these measures may be inadequate to evaluate whether this type of intervention is effective for its intended purpose[42]. An alternative outcome to explore in future studies is self-efficacy.

While stories can enhance recall of information, the advantages may be less apparent if the audience is highly motivated or educated[43]. In our sample, the education level was relatively high. Many narrative or story interventions have been developed and evaluated within populations of lower education and literacy levels, lower socio-economic status, and sometimes those with “a distrust of authorities.”[44] These populations are often the most difficult to reach[44], and may not have other information sources. 

### Future Research

Our experience leads us to make several recommendations for future research. Researchers need to clearly identify the purpose of the stories prior to development and evaluation. The purpose and timing of the intervention need to match the needs of the end-user[45], and should be identified through a systematic process. The outcomes of most importance will also vary by the needs of the end-users, as well as the clinical context. Careful consideration is needed for the study comparisons. Significant differences in this literature have more often been found when the intervention was compared against standard care or waitlist control, whereas fewer differences have been observed when compared to another active intervention[46].

This topic area presents a unique challenge in that the development and pilot testing [47] of the stories and how they are packaged is a critical step. There are a number of characteristics of the intervention that may influence its effectiveness, such as the readability and level of language, length and format, writing style, and, capacity for emotional engagement[43,44,48-52]. Another challenge is that there are numerous aspects of the interventions that can be varied, such as the medium of delivery (e.g., booklets, video, computer) and presentation (e.g., illustrations, images, colours, shape, and size)[49]. Researchers need to consider the optimal design of the intervention[51], and these attributes may shift in response to the audience, clinical condition, and end goal.

### Strengths and Limitations

We developed our intervention through an iterative process which involved pilot testing among healthcare professionals for content validity and focus groups of parents for appeal and readability[27]. We implemented a randomized trial representing the highest level of evidence for an intervention. Blinding was a challenge due to the nature of the intervention. We blinded the participants to the study hypothesis and the interventions being compared. Where possible, we used validated tools to measure outcomes. We conducted a separate qualitative study where we interviewed a sample of parents who received the stories; the methods and results have been reported elsewhere and should be considered in planning future research[40]. Despite the scientific rigour of the present study, numerous questions remain for future work in order to identify for whom and in what contexts narrative communication may be most effective[43]. 

## Conclusions

This randomized trial comparing story booklets with standard information sheets for parents of children attending the ED with croup showed no difference in the primary outcome of change in parental anxiety. The story group showed significantly greater decision regret and quicker time to resolution of symptoms; further research is required to substantiate these findings and their clinical significance. No differences were observed for the remaining outcomes. Potential reasons for lack of significant findings include choice of outcome, timing of outcome assessment, and disconnect between the nature of the intervention and the needs of the target audience. Finally, the story intervention may offer no advantage over standard information sheets; both study groups received interventions which overlapped in terms of content, thereby minimizing the relative impact of one intervention over the other. 

## Supporting Information

Checklist S1 **CONSORT Checklist.**
(DOC)

Protocol S1 **Study protocol.**
(PDF)
