# Supplementary material for: A Randomized Controlled Trial of Storytelling as a Communication Tool
Source: PLoS One. 2013 Oct 25;8(10):e77800. doi: 10.1371/journal.pone.0077800 (PMC3808406; doi:10.1371/journal.pone.0077800)
Supplement: Protocol S1 — Study protocol. (PDF) [file pone.0077800.s002.pdf]

HEALTH RESEARCH ETHICS BOARD
REQUEST FOR ETHICS REVIEW FORM

***Note – Please complete this form by following the *"Instructions for Completing the HREB Request for Ethics Review Form"*.**

SECTION A: GENERAL INFORMATION

**A1. Project Title**
Title of Project: A Randomized Controlled Trial of storytelling as a communication tool aimed at parents of children presenting to the emergency department with croup

**A2. Applicant Information**			
Name: Terry P. Klassen<br>Title: Professor and Chair<br>Department: Pediatrics			
Mailing Address: Room 8213, Aberhart Centre One, 11402 University Avenue			
City & Province:<br>Edmonton, AB	Postal Code:<br>T6G 2J3	Phone:<br>(780) 407-7084	Fax:<br>(780) 407-8538
E-mail Address: terry.klassen@ualberta.ca			
Signature:			Date:<br>May 9, 2007

**A3. Co-Applicant Information**			
Name: Lisa Hartling<br>Title: Assistant Professor<br>Department: Pediatrics			
Mailing Address: Room 9424, Aberhart Centre One, 11402 University Avenue			
City & Province:<br>Edmonton, AB	Postal Code:<br>T6G 2J3	Phone:<br>(780) 492-6124	Fax:<br>(780) 407-6435
E-mail Address: hartling@ualberta.ca			
Signature:			Date:<br>May 9, 2007

**A4. Authorizing Signature**	
Indication of Department Support for the Implementation of the Project.	
Name of Dept. Chair, Assoc. Dean of Research, or Supervisor:	
Title:	
Signature:	Date:

A3. Co-Applicant Information			
Name: Shannon Scott-Findlay<br>Title: Post-doctoral Fellow<br>Department: Pediatrics			
Mailing Address: Room 9432, Aberhart Centre One, 11402 University Avenue			
City & Province:<br>Edmonton, AB	Postal Code:<br>T6G 2J3	Phone:<br>(780) 492-5074	Fax:<br>(780) 407-6435
E-mail Address: Shannon.scott-findlay@ualberta.ca			
Signature:			Date:

**A5. Co-Investigators / Thesis Committee**		
Is this project for a graduate thesis? (X) Yes ( ) No<br>*If yes, please provide the names, departments, and phone numbers of your thesis committee.*		
Name:	*Department/Program:*	*Phone:*
Dr. Thierry Lacaze	Pediatrics	(780) 407-3783
Dr. Ted Bishop	English and Film Studies	(780) 492-7844
Dr. David Johnson	Pediatrics and Pharmacology & Toxicology, U of C	(403) 943-7507

**A6. Expedited Review**	
If the study procedures are ***LIMITED*** *to any of the following, please check (√):*	
	Analysis of blood, urine, or any other biological specimen already collected.
	Examination of patient, medical, or institutional records.
	Modification of a previously approved protocol (specify title and approval date):
	Secondary analysis of data.
	Use of biological specimens normally discarded.

**A7. Type of Investigation**			
Which one of the following best describes the type of investigation proposed? Check (√) more than one if appropriate.			
	Clinical Trial	√	Multi-centre Trial
	Drug Study		Pilot Study
	Epidemiological Study		Qualitative Study
	First Application in Humans		Technology Assessment / Development
	Sequel to Previously Approved Project (specify title and approval date):		
	Other (specify):		

**A8. Site of Research**	
Where will the research be conducted? Check (√) more than one if appropriate. Specify the area/department/program.	
Alberta Cancer Board Sites:	
	Cross Cancer Institute:
Capital Health Authority Sites:	
	Community Care and Public Health:
	Glenrose Rehabilitation Hospital:
	North Edmonton Community Health Centre:
	Royal Alexandra Hospital:
√	Stollery Children's Health Centre: Emergency Department
	Sturgeon Community Hospital and Health Centre:
	University of Alberta Hospital:
Caritas Health Group Sites:	
	Edmonton General Hospital:
	Grey Nuns Community Hospital and Health Centre:
	Misericordia Community Hospital and Health Centre:
University of Alberta Sites:	
	Specify (e.g. Corbett Clinic):
Other:	
√	Specify (e.g. Edmonton Public Schools, Subjects' homes):<br>Alberta Children's Hospital, Calgary
Letters of Support:	
(√) Pending	( ) Attached ( ) Not Applicable

**A9. Funding / Budget**	
How is the project funded? Please check (√) the appropriate box.	
	Funding approved; specify source(s):
√	Funding pending; specify source(s): CIHR Team Grant
	No external funding required.
Budget	
√	Please check here (√) that you have attached a budget summary. The summary must include details of investigator payments and recruitment incentives (if present). Please attach the budget as an appendix to the form.

**A10. Remuneration**	
Are any of the investigators involved receiving any directs personal remuneration or other personal or family financial benefits (either direct or indirect) for taking part in this investigation?	
	Yes. If so, append a letter detailing these activities. Please attach this letter to your budget summary.
√	No.

**A11. Safety Approvals**					
Please check (√) whether or not this study requires any of the following safety approvals. If a safety approval is needed, please indicate whether the approval documentation is pending or attached as an appendix to this form.					
Biohazardous Materials:					
√	Not Applicable		Pending		Attached
Electromechanical:					
√	Not Applicable		Pending		Attached
Health Protection Branch or Other Canadian Federal Agency:					
√	Not Applicable		Pending		Attached
Radiation:					
√	Not Applicable		Pending		Attached

SECTION B: DETAILS OF PROJECT

**Description of the Project**
B1. Provide a clear statement of the purpose and objectives of the project.<br>The purpose of this trial is to investigate storytelling as a tool to engage parents in communicating research and health information in order to affect parental anxiety and other outcomes. The primary objective is to evaluate the effectiveness of story booklets compared to standard information sheets in terms of reducing anxiety of parents who attend the emergency department with a child with croup. The secondary objective is to evaluate the effectiveness of story booklets compared to standard information sheets in terms of parental knowledge, satisfaction, decisional regret, and healthcare utilization and costs. The third objective is to use qualitative research methodologies to examine the reasons for which stories may or may not be an effective medium for transferring knowledge (both research evidence and experiential knowledge) to parents.
B2. State the hypotheses and/or research questions.<br>The principal research question is*:* Can we affect parental outcomes and resource use through stories that integrate research and health information with personal experience? The hypothesis is that stories, delivered through printed and illustrated story booklets, versus standard information sheets distributed in the ED, will produce different results in terms of parental anxiety, knowledge, satisfaction, and decisional regret; healthcare utilization patterns; and costs.
B3. Briefly summarize past human and/or animal research that has lead to this project.<br>Attending the ED is an anxiety-provoking experience for children and their parents. One of the major sources of parental anxiety is “uncertainty about what will happen at the hospital and unanswered medical questions”. Providing timely and useful information to parents can assist in managing their anxiety. Informing and preparing parents and children of what to expect is also linked to parent satisfaction, compliance, and cooperation during and after the ED visit. Research has demonstrated that standard written instructions are not as effective as more innovative methods of presenting information. Recently, there has been resurgence in the use of storytelling and narrative in medicine, for example in the areas of diagnostics, therapeutics, and the education of patients, students, and practitioners. A review of the literature showed that narrative is being used in many different forms, as well as for a wide variety of conditions and situations. However, few studies have evaluated narrative/stories in rigorous trials and among the trials that exist, there is variation in the purpose of the stories and target populations. Only one study involved a clinical population and addressed management of anxiety. This trial evaluated a 3-phase educational-behavioural intervention for children admitted to a PICU and their mothers which included reading and discussing a story about a young child who successfully copes with a stressful hospitalization. The intervention reduced parental anxiety (effect size of 0.32 at 1 month post-discharge), depression, and symptoms of post-traumatic stress disorder. Overall, the literature illustrates that storytelling is being sought as a tool to communicate with and influence patients or at-risk populations, however, the paucity of rigorous clinical research studies underscores the need for evidence to confirm or refute the value of this modality as a communication tool within the healthcare setting, and specifically in the context of pediatric care.

Description of Sample/Population	
B4. Describe the numbers and type(s) of subjects to be included. If appropriate, specify the number of subjects in each study group. Provide a rationale for the sample size and include sample size calculations where appropriate.<br>The target population is parents who are attending the pediatric emergency department with a child with croup. We plan to recruit 210 parents in each group (story booklets vs. standard information sheets). Our alternate hypothesis is that change in anxiety from beginning to end of the ED visit will be different for the group receiving story booklets versus the comparison group. In the absence of data specific to parents of children with croup attending the ED, we based our estimates for sample size calculations on previous research in similar clinical populations. We estimate that the initial anxiety level in both groups will be approximately 45 on the State Trait Anxiety Inventory Scale (STAI-S). Two studies of parents whose children were undergoing elective surgery reported anxiety levels of 45.97 and 44.76, respectively. We believe these estimates to be conservative; for instance, parents bringing their young (<2 years) febrile children to the ED scored 50.1; parents of hospitalized children requiring total parenteral nutrition showed baseline anxiety levels of 59.5; and parents of children admitted to the PICU showed levels of 52.8. In the latter study evaluating a multi-faceted intervention including storytelling, mothers in the intervention and control groups had average anxiety levels of 36 and 40, respectively, one-month post-discharge. Based on these findings, we hypothesize that parents in the story booklet group would return to a 'normal' level of anxiety following treatment (i.e., 36 or 37), while those in the comparison group will remain more anxious (i.e., 39 or 40). We conducted sample size calculations, using a two-sided, two sample t-test with a significance level of 0.05 and standard deviation of 10 (based on the cited studies), to detect a difference of 3 or 4 points on the STAI-S scale. This effect size (0.3 and 0.4 respectively) is comparable to previous research evaluating an intervention involving a story and written information provided to parents of hospitalized children. For 80% power, we will require 176 individuals per group for a 3-point difference in anxiety scores. We inflated our planned sample size by 20% (based on evidence regarding possible impact of contamination) (210 per group) to account for potential contamination and drop-outs.	
B5. List any subject inclusion/exclusion criteria.<br>Parents of children with a clinical diagnosis of croup will be eligible for study. Parents must also meet the following criteria: 1) have a telephone and be willing to be contacted for follow-up interviews; 2) fluent in English; 3) provide informed consent; 4) no prior visit to an ED during this episode of the disease; 5) no prior visit to an ED for another episode of croup during the study period. Parents will be excluded if: 1) stridor is due to another cause (e.g., bacterial tracheitis, presence of a supraglottic foreign body); 2) parent has previously been included in the study.	
B6. Please check (√) if any of the subjects who will be recruited fall into one or more of the following categories:	
√	Under 18 years of age
	Cognitively Impaired
	Residing in institutions (e.g. prison, extended care facility)
	Students

	Employees of researchers' organization
	Have language barriers (e.g. illiterate, not English-speaking, dysphasic)
	In another country

**Description of Research Procedures**
B7. Provide a summary of the design and procedures of the research. Provide details on the methods of data collection and data analysis, time commitment for the subjects etc. Please note that any and all study measures need to be appended to the copies of the research / grant proposal (e.g. questionnaire, interview guides, rating scales etc.).<br>This will be a randomized trial involving 2 sites (Stollery Children's Hospital and Alberta Children's Hospital in Calgary). Consenting parents of children with croup will be randomized to receive a story booklet or a standard information sheet. They will receive the intervention at the beginning of the ED visit. The parents will be aware that we are evaluating different approaches to managing children in the ED, but will be unaware of the specific study hypotheses; they will be assured that the study will not affect the medical management of their child. Data collectors and data analysts will be blind to the study group of the participants. Because of the nature of the intervention, the research nurse and the other ED personnel involved in the study will not be blind to the study groupings of the participants. Parents will be interviewed upon entry into the study and on discharge from the ED. Parents will be contacted by telephone at 1 and 7 days following their visit to the ED. Parents will also be contacted by telephone 1-year following their ED visit to determine whether they have experienced other episodes of croup either with the child with whom they attended the ED or with other children they have, how they managed the other episodes (e.g., visit to ED, managed at home), and the resources they used (e.g., medication). A convenience sample of 30 parents who received the story booklets will be invited to participate in a 30-60 minute semi-structured, telephone interview as part of the qualitative objective. At the end of the 7-day follow-up, the parents will be asked if they consent to be invited to participate in the qualitative interview. If the parents consent, an interview time will be coordinated occurring approximately two weeks after their visit to the ED.
B8. Which treatments or procedures are additional to those required for standard patient care?<br>Parents are sometimes given a patient information sheet on discharge from the emergency department. In this study, every parent will receive information but the format will vary depending on the study group to which they are allocated. Patients will receive standard medical management for their presenting illness at the discretion of the attending emergency department staff.
B9. If the procedures include a blind, under what conditions will the code be broken and what provisions have been made for this? Who will have the code?<br>The emergency department staff will know what intervention the parents have received. This is a non-invasive intervention.

Obtaining Consent
B10. Clearly detail who will be recruiting subjects and obtaining consent, and the procedures for doing this. If appropriate specify whether subjects will be randomly assigned to groups before or after consent has been attained.<br>The research nurse in the ED will recruit participants and obtain informed consent. Immediately after consent, the research nurse will collect demographic information and participants will complete a questionnaire on anxiety. The research nurse will assess the severity of the child's illness using the Westley Croup Score. The research nurse will open the next allocation envelope and document which intervention the parent received. On discharge from the ED, participants will complete another short questionnaire to assess parental anxiety. After the ED visit, the enrolment and consent forms will be faxed to the study coordinator who will assign the case to a centrally located and trained interviewer who will then contact the parent at 24 hours after the ED visit. The initial telephone interview will take approximately 15 to 20 minutes. Subsequent interviews will be conducted at 7 days and 1 year after the ED visit, with an optional qualitative research interview at 14 days post-ED visit.
B11. Specify methods for dealing with groups identified in #B6. If the subjects are not able/competent to give fully informed consent, who will consent on their behalf?<br>We will be recruiting parents of children with croup. Although we are not specifically targeting parents who are less than 18 years old, it is possible that some of the parents attending the emergency department with a child with croup may be less than 18 years old. These individuals must be competent to give fully informed consent in order to participate in the study.
B12. If the subjects will be offered compensation for participating in the research, provide details. Specify the amount, what the compensation is for, and how payment will be determined for subjects who do not complete the study.<br>No compensation will be offered to study subjects for participation in the study.
B13. Do any of the procedures include the use of deception or partial disclosure of information to subjects? If yes, provide rationale for the deception or partial disclosure. Describe the procedures for (a) debriefing the subjects and (b) giving them a second opportunity to consent to participate after debriefing.<br>The parents will be aware that we are evaluating different approaches to managing children in the ED, but will be unaware of the specific study hypotheses; they will be assured that the study will not affect the medical management of their child. This is to ensure that the parents are blind to the intervention. Given that it is an educational intervention as opposed to a drug, blinding is much more challenging. Based on reviewers' comments to our CIHR application, this is the option we have chosen. We will debrief study participants at the end of the data collection period. This is a non-invasive intervention and will not affect the medical management that the child receives in the emergency department.

Recruitment Aids/Information Letters/Consent Forms
B14. Are you planning to use any recruitment aids such as posters, newspaper advertisements, radio announcements, or letters of invitation? If so, please indicate the reading level of each aid and check (√) if it has been attached to the form as an appendix.

Recruitment Aid #1 – Specify (e.g. poster, letter etc.):					
√	Not Applicable		Reading Level		Attached
Recruitment Aid #2 – Specify:					
√	Not Applicable		Reading Level		Attached
Information Letter #1 – Specify (e.g. Letter for interviews, focus groups etc.):					
√	Not Applicable		Reading Level		Attached
Information Letter #2 – Specify:					
√	Not Applicable		Reading Level		Attached
Consent Form #1 – Specify (e.g. Consent for interview, focus group etc.):					
	Not Applicable	11.8	Reading Level		Attached
Consent Form #2 – Specify:					
	Not Applicable	11.4	Reading Level		Attached
B15. What steps have been taken to make the recruitment aids, information letters, and consent forms comprehensible to the person(s) giving consent?<br>The consent forms have been written with a lay audience in mind, using simple language and sentence structures.					

**Risks and Benefits**
B16. What are the benefits of the proposed research for the subject and/or for scientific knowledge in general?<br>The results of this trial will be an important advancement for knowledge translation (KT) in understanding whether storytelling is an effective means for transferring information to patients and their families. If found to be effective, story booklets may be distributed in lieu of standard information sheets in the pediatric EDs of the two sites involved in the trial (Stollery Children's Hospital in Edmonton [SCH] and Alberta Children's Hospital in Calgary [ACH]). The results will be disseminated through various means, including publication in peer-reviewed journals, presentation at scientific conferences, and communications with key stakeholders. This trial is one part of a multi-phase research program funded under the CIHR Team in Pediatric Emergency Medicine. The results will inform subsequent steps including the development and evaluation of stories in other clinical areas and for specific cultural groups, as well as the development of other story-based communication tools (e.g., websites, computer games).
B17. What adverse effects may result from the research? How will adverse effects be dealt with? Please note that adverse effects are not limited to physical risks, but include psychological, emotional, and spiritual risks as well.<br>Study participation presents no known risks to the participants, will pose no significant inconvenience or cost, and will not submit the participants to any pain or suffering. Patients will receive standard medical management at the discretion of the attending physician.

**Privacy and Confidentiality**
B18. What steps will be taken to respect the privacy of the subjects and protect confidential data?<br>The data collection forms will not contain any personal identifiers. We will use a study identification number and initials to identify the study group to which the parent has been assigned and link the data collection forms to the same individual. The data collection forms will be kept in a locked filing cabinet in the research office of the principal investigator for 7 years following the trial. Data entered into the computer will have no confidential information; a study identification number will be used to link data. Results will be reported in a way that does not identify individual parents.
B19. Identify any agencies or individuals who will have access to confidential data now or in the future.<br>The individuals who will have access to confidential data are the research nurse recruiting the parents in the emergency department, the telephone interviewers, and the co-investigator who will be performing the qualitative interviews on a subsample of parents. The principal investigator (Terry Klassen), co-applicant (Lisa Hartling), statistician (Ben Vandermeer) and health economist (Gillian Currie) listed on the project will have access to the raw data but this data set will be stripped of personal identifiers.
B20. Do you anticipate any secondary analysis of the data? Please note that any secondary analysis requires further research ethics approval.<br>We do not anticipate any secondary analysis of the data.
